# Ultra-Widefield Fluorescein Angiography-Based Targeted Retinal Photocoagulation versus Panretinal Photocoagulation in Proliferative Diabetic Retinopathy: A Randomized Controlled Trial

**DOI:** 10.12669/pjms.41.10.12535

**Published:** 2025-10

**Authors:** Imran Ahmad, Yousaf Jamal Mahsood

**Affiliations:** 1 Imran Ahmad, (MBBS, MRCS.Ed, FICO, FACS, FCPS, FCPS-Vitreoretina, MHPE) Associate Professor, Department of Ophthalmology, Khyber Medical College, Khyber Teaching Hospital, Peshawar, Pakistan; 2 Yousaf Jamal Mahsood, (MBBS, MHR, MRCSEd, FICO, FRCS, FCPS) Associate Professor, Department of Ophthalmology, Khyber Girls Medical College, Hayatabad Medical Complex, Peshawar, Pakistan

**Keywords:** Diabetic retinopathy, Fluorescein angiography, Laser photocoagulation, Panretinal photocoagulation, Targeted retinal photocoagulation

## Abstract

**Objective:**

To compare the safety and efficacy of ultra-widefield fluorescein angiography-based targeted retinal photocoagulation with panretinal photocoagulation for the treatment of proliferative diabetic retinopathy.

**Methodology:**

This randomized controlled trial was conducted from September 1, 2022, to February 28, 2024. Patients with proliferative diabetic retinopathy were randomly allocated to targeted retinal photocoagulation (TRP) and panretinal photocoagulation (PRP). In TRP group, the laser was applied only to the ischemic area demarcated by ultra-widefield fluorescein angiography, whereas in PRP group, the conventional PRP laser photocoagulation protocol was followed. Data was gathered for the treatment effects on best corrected visual acuity (BCVA), central macular thickness (CMT), mean deviation (MD) and visual field index (VFI).

**Results:**

A total of 120 participants were recruited during the study period, with 60 in each group. The mean age was 65.66 ± 9.33 years with 64 (53.3%) males. There was no statistically significant difference between the two groups at baseline and after the treatment for BCVA, CMT, MD and VFI. When compared within the groups, CMT improved from baseline which was statistically significant (p=0.002) in TRP group. The PRP group also showed significant improvement in CMT from baseline (p=0.01), but MD and VFI worsened significantly from the baseline (p<0.001).

**Conclusions:**

Targeted retinal and panretinal photocoagulation are equally effective in reversing proliferative diabetic retinopathy, but panretinal photocoagulation negatively affects mean deviation and visual field index.

## INTRODUCTION

Diabetic retinopathy (DR) is a leading cause of preventable blindness worldwide and its prevalence is of particular concern in developing countries like Pakistan, where the incidence of diabetes is rapidly increasing. In Pakistan, an estimated 26.7% of the adult population is living with diabetes and studies suggest that approximately one-third of these individuals may develop some form of DR.^1^,^2^ As the duration of diabetes and poor glycemic control are the major risk factors, the burden of DR is expected to rise, posing a major public health challenge. Proliferative diabetic retinopathy (PDR), the advanced stage of the disease, contribute for most diabetes-related blindness and significantly affects the quality of life and socioeconomic well-being of affected individuals. Limited access to specialized ophthalmic care, inadequate screening programs and delayed interventions exacerbate this problem, highlighting the urgent need for effective and accessible treatment strategies. Addressing the rising burden of DR in Pakistan is crucial for reducing the prevalence of vision loss and improving health outcomes in the growing diabetic population. Diabetic retinopathy and diabetic macular edema are the most common causes of visual loss in diabetic patients.^3^

The recommended treatment for PDR includes retinal laser photocoagulation and intravitreal anti-Vascular Endothelial Growth Factors (VEGF) injections.^4^ The panretinal photocoagulation (PRP) is the commonly used procedure recommended by the diabetic retinopathy study (DRS)^5^ in 1976 and is still used as the gold standard in developing countries due to poor socioeconomic conditions, lack of affordability and non-availability of anti-VEGF injections.^6^ Early Treatment of Diabetic Retinopathy Study (ETDRS) recommended that retinal photocoagulation decreases the chances of visual loss and improves the regression of neovascularization in patients with PDR.^7^

The VEGF produced by the ischemic retina are responsible for vascular leakage, angiogenesis and complications of proliferative diabetic retinopathy.^8^ The mechanism of PRP is ablation of the ischemic retina, reducing the VEGF drive, with a decrease in vascular leakage and angiogenesis.^9^,^10^ Retinal photocoagulation is not a “one-and-done” procedure but is to be repeated again and again depending upon the severity, recurrence of retinopathy and control of risk factors.^11^ Despite its beneficial effects in the regression of PDR, PRP is reported to result in visual loss, increase in central macular thickness, constriction of the visual field and loss of contrast sensitivity.^12^,^13^ These complications are particularly concerning in PDR patients, where maintaining overall visual function is crucial for quality of life, especially in regions like Pakistan, where healthcare access and socioeconomic support are limited.

These limitations demand a more precise strategy, such as targeted retinal photocoagulation (TRP). TRP is based on ultra-widefield fluorescein angiography, in which the laser is targeted to the area of retinal capillary non-perfusion as demarcated angiographically sparing the well-perfused retina.^14^ By photocoagulating only the targeted ischemic retina, VEGF release from the non-perfused area will be reduced without harming the normal retina and, consequently will have less effect on vision and visual field loss as compared to PRP. Further laser can be augmented depending on the area of non-perfusion or failure of retinopathy regression. Given the higher incidence of diabetes and limited access to advanced retinal care in Pakistan, the ability of TRP to offer similar efficacy with fewer side effects makes it a promising alternative to the conventional PRP. Exploring the safety, efficacy and long-term benefits of TRP is essential for optimizing treatment strategies for PDR, particularly in low-resource settings where minimizing vision loss and preserving the quality of life are paramount. However, there is currently limited high-quality evidence that directly compares TRP with PRP in terms of both safety and efficacy.

This research is important because it could inform clinical practice by potentially offering a treatment that achieves similar or superior outcomes to PRP, while minimizing the adverse effects that contribute to long-term visual morbidity in patients with PDR. If TRP proves to be safer and equally or more effective, it could become a preferred treatment modality, particularly for patients who are at risk of significant vision loss. Thus, this study addresses an unmet need for optimizing diabetic retinopathy treatment by balancing therapeutic benefits with visual function preservation. This study aimed to assess whether selective targeting of ischemic retinal areas with TRP offers a superior balance of therapeutic benefits and fewer adverse outcomes compared to the more widespread retinal ablation induced by PRP.

## METHODOLOGY

This study was carried out from September 1, 2022, to February 28, 2024. With a 95% confidence level, 5% margin of error and an 8.6%^15^ prevalence of proliferative diabetic retinopathy, the estimated sample size was 120. The participants were randomly allocated by the lottery method into two groups: targeted retinal photocoagulation (TRP) and panretinal photocoagulation (PRP).

### Ethical Approval:

The study was initiated after obtaining approval from the Institutional Research and Ethical Board (IERB) through notification No. 306/DME/KMC; dated April 19, 2024. This was a randomized controlled trial (ClinicalTrials.gov ID: NCT06653361) and was included in the trial registry. Written informed consent was acquired from each participant and the declaration of Helsinki’s principles were followed throughout this study.

### Inclusion Criteria:

Participants with proliferative diabetic retinopathy (PDR) with neovascularization on the disc or elsewhere in the retina were examined clinically by a consultant ophthalmologist to confirm the diagnosis and were enrolled in the study. Each participant underwent a thorough examination that included fundoscopy with a super-field lens, slit-lamp anterior segment examination, intraocular pressure (IOP) using Goldman applanation tonometry and best-corrected visual acuity (BCVA). Spectral-domain Optical coherence tomography (Spectralis Heidelberg OCT-2) was performed on all participants to check for central macular thickness (CMT). Pre-treatment and post-treatment Humphrey visual fields (30-2) were assessed using the Swedish Interactive Threshold Algorithm (SITA) fast strategy in all patients.

### Exclusion Criteria:

Participants with a history of retinal photocoagulation, vitreoretinal surgery, tractional retinal detachment, epiretinal membrane, vitreomacular traction, glaucoma, uveitis, media opacities such as cataract, corneal opacity, vitreous hemorrhage, or history of intravitreal anti-VEGF injections were excluded from the study. Participants with abnormal renal function, pregnancy, or sensitivity to fluorescein dye were also excluded from the study to avoid fluorescein-related complications.

In both groups patients underwent optos (California) ultra-widefield fluoresceine angiography. In this procedure, 5 ml of 10% sodium fluorescein was injected intravenously into the antecubital vein for 5-6 seconds and fundus photographs were taken after eight seconds of injection and then every second until 30 seconds, one minutes, two minutes and five minutes. In TRP group, demarcating the non-perfused retina through ultra-widefield angiography, an argon green laser (Vitra-2, 532 nm Quantel Medical) was applied only to the non-perfused retina (minimum one disc diameter area of non-perfusion) using sufficient power to blanch the retinal tissues, with a spot size of 200-300 microns at the 0.1-second interval with a power of 200 - 400mW. In the PRP group, the conventional PRP was done using an argon green laser to the retina outside the temporal vascular arcade and the peripheral retina using a spot size of 200 - 300 microns at 0.1-second intervals with a power of 200 - 400mW to get blanching of retinal tissues. Both TRP and PRP were performed in a single setting. A minimum of two months after photocoagulation treatment, FFA was repeated to check for neovessels and were again assessed for BCVA, CMT on OCT, Humphrey analyzer for changes in visual field mean deviation (MD) and visual field index (VFI) and fundoscopy for regression of proliferative diabetic retinopathy.

Participants were enrolled in the outpatient department of ophthalmology of Khyber Teaching Hospital, Peshawar. All pre- and post-treatment data were recorded using a proforma. Data were analyzed using SPSS software version 26. Best-corrected visual acuity (BCVA), neovascularization regression, central macular thickness (CMT) on OCT and visual field changes by comparing mean deviation (MD) and visual field index (VFI) values before and after therapy were among the parameters to be measured. While frequencies were computed for qualitative data, the mean, median and standard deviation were computed for quantitative data. For quantitative data comparison, the student’s t-test was applied. The means of the groups were compared using an independent sample t-test, while comparisons within the group were made using a paired t-test. A significance level of P < 0.05 was established.

## RESULTS

**Fig.1:**
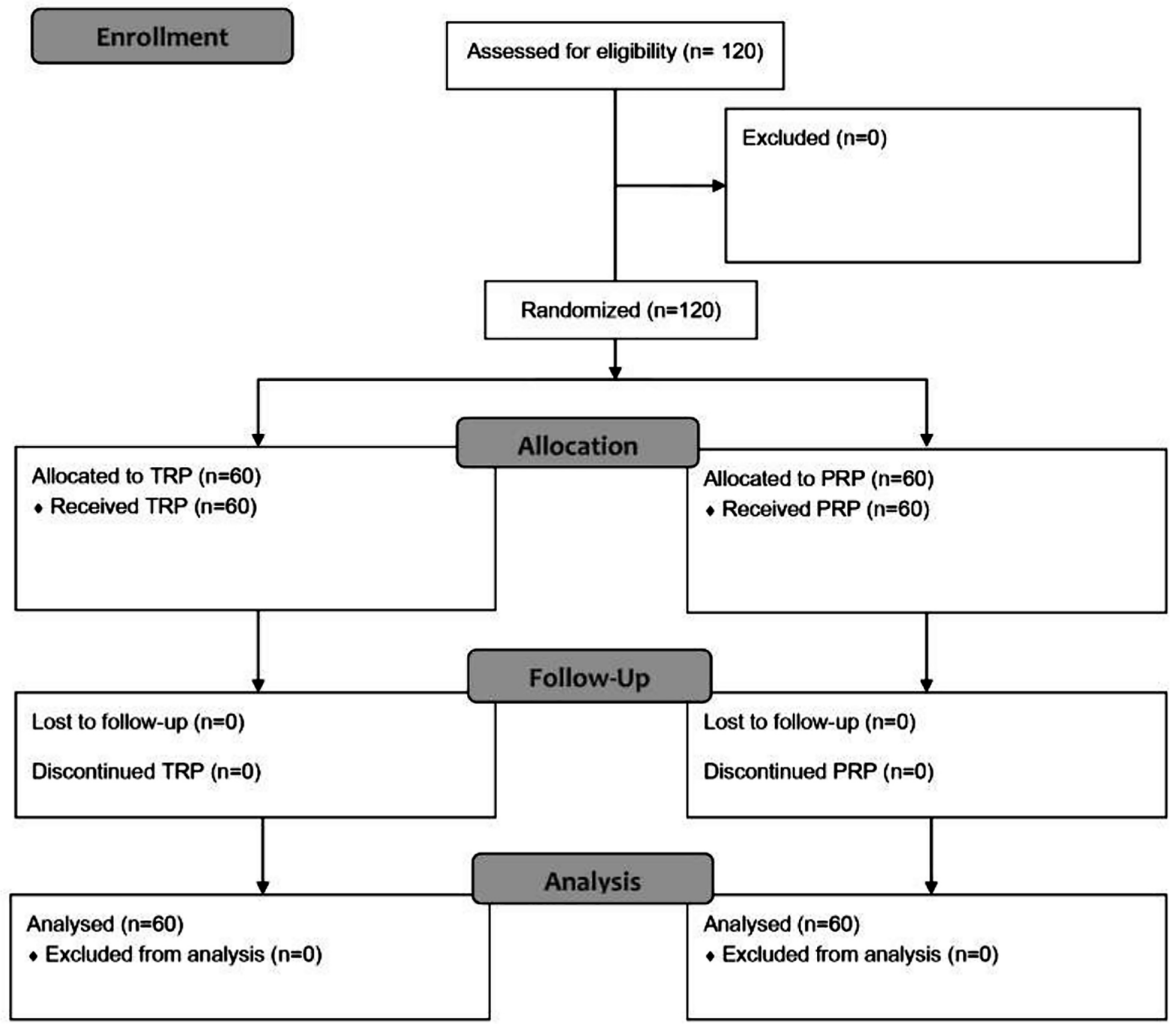
CONSORT 2010 Flow Diagram.

With 60 participants in each group, the study had 120 participants in total. The CONSORT 2010 flow diagram is shown in Fig.1. The mean age of the participants was 65.66 ± 9.33 years and 64 (53.3%) were male. There were no statistically significant differences in BCVA, CMT, MD and VFI between the groups at baseline (p=0.15 for BCVA, 0.99 for CMT, 0.82 for MD and 0.98 for VFI) and is shown in Table-I.

**Table-I:** Baseline demographics of the study participants.

Parameters	Targeted retinal photocoagulation	Panretinal photocoagulation	P-value*
Gender, n (%)	Male = 64 (53.3)	Female = 56 (46.7)	
BCVA (LogMAR)	0.572 ± 0.26	0.60 ± 0.24	0.15
CMT (microns)	350.82 ± 73.75	362.93 ± 76.32	0.99
MD (dB)	-6.42 ± 2.62	-6.76 ± 2.74	0.82
VFI (%)	84.88 ± 5.66	83.63 ± 5.77	0.98

n=frequency, %=percentage.

* Independent sample t-test was applied.

Table-II shows a comparison between the groups after minimum of two months endpoint after treatment, but the difference was not statistically significant (p=0.57 for BCVA, 0.12 for CMT, 0.24 for MD and 0.16 for VFI).When compared within the groups for before and after treatment as shown in Table-III, CMT improved from 350.82 ± 73.75 to 329.07 ± 43.19 microns which was statistically significant (p=0.002) while there was no statistically significant difference noted in BCVA, MD and VFI in TRP group. In PRP group, CMT improved from 362.93 ± 76.32 to 339.47 ± 54.42 microns which was statistically significant (p=0.01) while MD (-6.76 ± 2.74 to -9.37 ± 2.70, p<0.001) and VFI (83.63 ± 5.77 to 79.48 ± 5.028, p<0.001) worsened in this group that was statistically significant.

**Table-II:** Comparison of post-treatment parameters between the two groups.

Parameters	Targeted retinal photocoagulation	Panretinal photocoagulation	P-value*
BCVA (LogMAR)	0.54 ± 0.21	0.57 ± 0.24	0.57
MD (dB)	-7.30 ± 4.45	-9.37 ± 2.70	0.24
VFI (%)	84.78 ± 6.05	79.48 ± 5.028	0.16
CMT (microns)	329.07 ± 43.19	339.47 ± 54.42	0.12

%=percentage, BCVA=Best Corrected Visual Acuity, LogMAR=Logarithm of the Minimum Angle of Resolution, CMT=Central Macular Thickness, MD=Mean Deviation, dB=decibel, VFI=Visual Field Index

* Independent sample t-test was applied.

**Table-III:** Comparison of pre- and post-treatment parameters within the groups.

Parameters	Targeted retinal photocoagulation				Panretinal photocoagulation			
	Pre- treatment	Post-treatment	95% CI	p-value^±^	Pre-treatment	Post-treatment	95% CI	p-value^±^
BCVA (LogMAR)	0.57	0.54	-0.05 - 0.11	0.47	0.60	0.57	-0.55 - 0.12	0.47
CMT (microns)	350.82	329.07	8.32 - 35.17	0.002	362.93	339.47	4.65 - 42.28	0.01
MD (dB)	-6.42	-7.30	-0.25 - 2.01	0.12	-6.76	-9.37	2.23 - 2.99	< 0.001
VFI (%)	84.75	84.78	-0.55 - 0.49	0.9	83.63	79.48	3.36 - 4.94	< 0.001

CI=confidence interval, BCVA=Best Corrected Visual Acuity, LogMAR=Logarithm of the Minimum Angle of Resolution, CMT=Central Macular Thickness, MD=Mean Deviation, dB=decibel, VFI=Visual Field Index, %=percentage.

± Paired sample t-test was applied.

Regression of neovascularization was documented in 89 (74.16%) patients, whereas neovessels were still present after laser photocoagulation in 31 (25.85 %) patients. In the TRP group, 44 (73.33%) participants achieved regression of neovascularization, whereas in the PRP group, in 45 (75 %) participants achieved regression of neovascularization. Thirteen (10.83%) patients developed complications of diabetic retinopathy, six in the TRP group and seven in the PRP group.

## DISCUSSION

This study assessed whether selective targeting of ischemic retinal areas with TRP provided a superior balance of therapeutic benefits and fewer adverse outcomes compared to the more widespread retinal ablation induced by PRP. The findings demonstrate that although both treatment options had no significant effect on final best-corrected visual acuity, TRP successfully minimized treatment-related complications related to visual field parameters, highlighting its potential as a safer alternative to conventional PRP.

In our study, both treatment options were equally effective at reducing CMT in patients with PDR. However, the visual acuity did not improve in the study participants. Despite reduced central macular thickness after laser treatment, the lack of visual improvement may result from permanent photoreceptor damage, chronic edema, macular ischemia, disorganization of the retinal inner layers (DRIL), inner retinal damage, residual diffuse edema, subretinal fluid, epiretinal membrane formation, persistent cystoid changes, or long-standing neuro-visual pathway alterations. One of the reasons for this could be persistent macular edema due to the shorter follow-up period in this study. Persistent treatment and longer follow-up may influence vision. The same observations were made by Nikkhah H et al in their study in which they concluded that there is no significant difference in vision and CMT between the two laser treatment modalities.^16^

PRP is associated with many complications including exaggeration of macular edema, loss of visual field, visual acuity and night vision. Soman M in their study noted the side-effects of visual field loss with PRP in PDR patients.^17^ Visual field loss is more associated with PRP as laser application is not based on ultra-widefield angiography but is applied to normal retina along with ischemic retina as suggested by Huang CX et al in their study.^18^ We applied TRP to the ischemic area demarcated by Optos ultra-wide retinal angiography. The same treatment strategy was suggested by Cai S and Liu TA in their study on the use of widefield angiography in DR patients for retinal photocoagulation.^19^ We took two parameters i.e. visual field index (VFI) and mean deviation (MD) of visual fields affected by laser photocoagulation.

In our study, the MD and VFI were significantly affected in PRP group whereas it was insignificantly affected in TRP group. The loss of significant visual field after PRP was also discussed by Bro T and Anderson J, who stated that, along with PRP, retinal ischemia is also responsible for visual field loss in diabetic patients after laser.^20^ There are variable reports mentioned in the literature on the effect of retinal laser on the visual field, as stated in the study by Baptista PM in their study stating that there is no consensus on visual field loss after retinal laser, some showing no effect of laser on visual fields, whereas other studies showed a significant effect.^12^ In our study, both MD and VFI were not significantly affected after TRP in PDR, showing that TRP is better than PRP in preserving visual fields after retinal photocoagulation.

The regression of neovascularization, which is a sign of treatment efficacy of PDR, was achieved in both groups, showing that ultra-widefield angiography-based targeted retinal photocoagulation is equally effective to that of PDR (73.33% in TRP vs. 75% in PRP group). A study by Faheem and Helmy stated that TRP is more effective than PRP in the regression rate of PDR (75% vs. 90%).^14^ However, we did not observe such a difference in our investigation, and they used a small sample size of 20 PDR patients.

### Strength & Limitation:

Our study’s strength is its randomized trial design and rather large sample size. However, the shorter follow-up duration and single-center study design are the main limitations. By involving more centers and recruiting more participants and a longer follow-up duration, we will be able to arrive at a definitive conclusion on the superiority of these procedures over each other. This may change the treatment protocols in the future.

## CONCLUSION

Targeted Retinal Photocoagulation is an effective treatment for proliferative diabetic retinopathy with an efficacy like that of panretinal photocoagulation. TRP can be used as an alternative treatment option for PRP, with the added advantage of less effect on the mean deviation of the visual field and visual field index compared to PRP.

### Authors’ Contribution:

**IA:** Contributed to the concept, design, literature search, data acquisition, manuscript preparation, manuscript editing, final approval of manuscript and agreed to be accountable.

**YJM:** Contributed to the design, literature search, data analysis, statistical analysis, manuscript preparation, manuscript editing, final approval of manuscript and agreed to be accountable.
